# Brain activation by a VR-based motor imagery and observation task: An fMRI study

**DOI:** 10.1371/journal.pone.0291528

**Published:** 2023-09-27

**Authors:** João D. Nunes, Athanasios Vourvopoulos, Diego Andrés Blanco-Mora, Carolina Jorge, Jean-Claude Fernandes, Sergi Bermudez i Badia, Patrícia Figueiredo

**Affiliations:** 1 INESC TEC - Institute for Systems and Computer Engineering, Technology and Science, and Faculty of Engineering, University of Porto, Porto, Portugal; 2 Institute for Systems and Robotics - Lisboa, and Department of Bioengineering, Instituto Superior Técnico, Universidade de Lisboa, Lisbon, Portugal; 3 Faculdade de Ciências Exatas e da Engenharia, N-LINCS Madeira — ARDITI, Universidade da Madeira, Funchal, Portugal; 4 Central Hospital of Funchal, Physical Medicine and Rehabilitation Service, Funchal, Portugal; University of Pittsburgh School of Medicine, UNITED STATES

## Abstract

Training motor imagery (MI) and motor observation (MO) tasks is being intensively exploited to promote brain plasticity in the context of post-stroke rehabilitation strategies. This may benefit from the use of closed-loop neurofeedback, embedded in brain-computer interfaces (BCI’s) to provide an alternative non-muscular channel, which may be further augmented through embodied feedback delivered through virtual reality (VR). Here, we used functional magnetic resonance imaging (fMRI) in a group of healthy adults to map brain activation elicited by an ecologically-valid task based on a VR-BCI paradigm called NeuRow, whereby participants perform MI of rowing with the left or right arm (i.e., MI), while observing the corresponding movement of the virtual arm of an avatar (i.e., MO), on the same side, in a first-person perspective. We found that this MI-MO task elicited stronger brain activation when compared with a conventional MI-only task based on the Graz BCI paradigm, as well as to an overt motor execution task. It recruited large portions of the parietal and occipital cortices in addition to the somatomotor and premotor cortices, including the mirror neuron system (MNS), associated with action observation, as well as visual areas related with visual attention and motion processing. Overall, our findings suggest that the virtual representation of the arms in an ecologically-valid MI-MO task engage the brain beyond conventional MI tasks, which we propose could be explored for more effective neurorehabilitation protocols.

## Introduction

Stroke is a leading cause of adult long-term disability [1]. Despite significant progress being made in post-stroke rehabilitation, there is still the need for further improvement of current rehabilitation strategies and their outcomes, as most survivors must cope with some degree of disability or loss of independence in activities of daily living (ADL) [2, 3]. Recovery after stroke implies reorganization of the cortex to compensate for the lesioned area. This is possible through neuroplasticity mechanisms, whereby the brain learns and reorganizes itself to compensate for lost functions [4]. Unfortunately, a considerable fraction of patients suffering from strokes affecting their motor function cannot fully benefit from current rehabilitation strategies due to factors such as a low level of motor control, reduced range of motion, pain, or fatigue[5].

Interestingly, research has shown that patients with severe stroke may benefit from motor imagery (MI) and/or motor observation (MO) training through the use of brain-computer interfaces (BCI’s). BCI’s can establish an alternative non-muscular channel between the patient’s brain activity and a computer, providing neurofeedback in a closed-loop. This can be used to strengthen key motor pathways that are thought to help promote brain plasticity mechanisms even in the absence of explicit movement [6–9]. The efficacy of such MI/MO BCI training systems for neurorehabilitation strongly depends on the ability of the MI/MO tasks to elicit the desired patterns of motor-related brain activation [10, 11]. In particular, the activation of the mirror neuron system (MNS) would be key to unravel the potential of MI/MO training systems for neurorehabilitation [12–15].

A growing body of research evidences that concurrent MI and MO might be superior to either condition alone in eliciting the desired brain activity [16–21]. However, the optimal type of task and respective instructions for MI / MO interventions remain to be clarified. In particular, some experimental paradigms present conflicts between the observed and imagined actions, such that the reported brain activity may include activation related with compensatory mechanisms [21]. Importantly, technological solutions based on virtual reality (VR) are increasingly adopted in post-stroke rehabilitation, and they have the potential to enhance the effectiveness of BCI approaches by providing more ecologically valid feedback on MI/MO performance [22–24]. We have previously developed a VR-based MI-MO task targeting the upper limbs for stroke rehabilitation—NeuRow [25]. It consists in performing MI of rowing with the left or right arm (i.e., MI), while observing the corresponding movement of the virtual arm of an avatar (i.e., MO), on the same side, in a first-person perspective. We have shown that such a VR-based task involving the consistent combination of MI and MO may be more powerful than conventional abstract MI tasks such as the ones based on the Graz-BCI paradigm [24, 26, 27]. However, the underlying brain activation remains to be investigated.

The neural correlates of MI and MO have been previously investigated, in particular regarding their relation with the brain regions recruited by motor execution tasks [28, 29]. However, there is some degree of heterogeneity in what regards combined MI-MO tasks, specially considering the wide variety of existing interventional protocols [9]. Importantly, only a few studies employ ecologically-valid scenarios, such as activities of daily living or embodied feedback with the use of VR. For example, a previous study showed that motor execution and the observation of virtual objects produced activation in areas of the MNS; however, the task did not involve ecologically-valid feedback nor embodied motor observation, rather a 2D paddle [30]. In another study, visual feedback from a VR-world (RGS) was used during both MI and MO, but the different task conditions took place in separate scanning sessions limiting direct comparisons [31]. Moreover, although the virtual environment used in this study (RGS) was previously designed for upper limb rehabilitation and has a good degree of ecological validity, it was not designed for neurofeedback nor MI-based BCI systems [32]. Finally, one study showed that MI additionally to MO recruited motor areas more than MO alone [33]. In this case, experimental conditions were designed to match as closely as possible the video therapy sessions involving goal transitive motor acts. However, no comparison with the more conventional MI task only was performed. To date, no study has yet directly compared brain activation with an ecologically-valid MI-MO task and directly compared it with a conventional MI-only task and with overt motor execution.

Here, we recruit a cohort of healthy adults and use functional magnetic resonance imaging (fMRI) to map brain activation elicited by the MI-MO task (NeuRow), and compare it with a commonly used abstract MI task based on the Graz BCI paradigm as well as overt motor execution. We aim to 1) map task-specific brain activation patterns; and 2) evaluate differences in brain activation patterns between tasks.

## Materials and methods

### Participants

A group of healthy right-handed participants was recruited (mean age 38.4 ± 13.2 years). The experimental protocol was designed in collaboration with the local healthcare system of Madeira, Portugal (SESARAM), and approved by the scientific and ethic committees of the Central Hospital of Funchal with approval reference number: 21/2019. The recruited cohort was assessed for their ability to perform kinesthetic motor imagery through the Kinesthetic and Visual Imagery Questionnaire (KVIQ) [34]. Furthermore, before each scan, and with the help of an occupational therapist, participants were asked to rehearse the visual and kinesthetic experience of moving their arms from the first-person perspective. Finally, a written informed consent was obtained from each participant upon recruitment, in accordance with the 1964 Declaration of Helsinki.

### Image acquisition

Imaging was carried out on a 3T GE Signa HDxt MRI scanner (General Electrics Healthcare, Little Chalfont, United Kingdom) using a 12-channel head coil. fMRI data were acquired using a multi-slice 2D gradient-echo EPI sequence (TR/TE = 2500/30 ms, voxel size = 3.75x3.75x3.00 mm^3^, flip angle = 90°, and FoV = 240x240 mm^2^). For co-registration purposes, whole-brain structural images were also acquired using a T1-weighted 3D Fast Spoiled Gradient-Echo (FSPGR) sequence (TR/TE = 7.8/3.0 ms, voxel size = 1.00x1.00x0.60 mm^3^). The visual stimuli were delivered through a specialized MR-compatible video visor (VisuaStim, Resonance Technology, Inc.) at a resolution of up to 800 × 600 pixels, at 60Hz refresh rate, and synchronized with the console computer.

### Experimental paradigm

**Fig 1:**
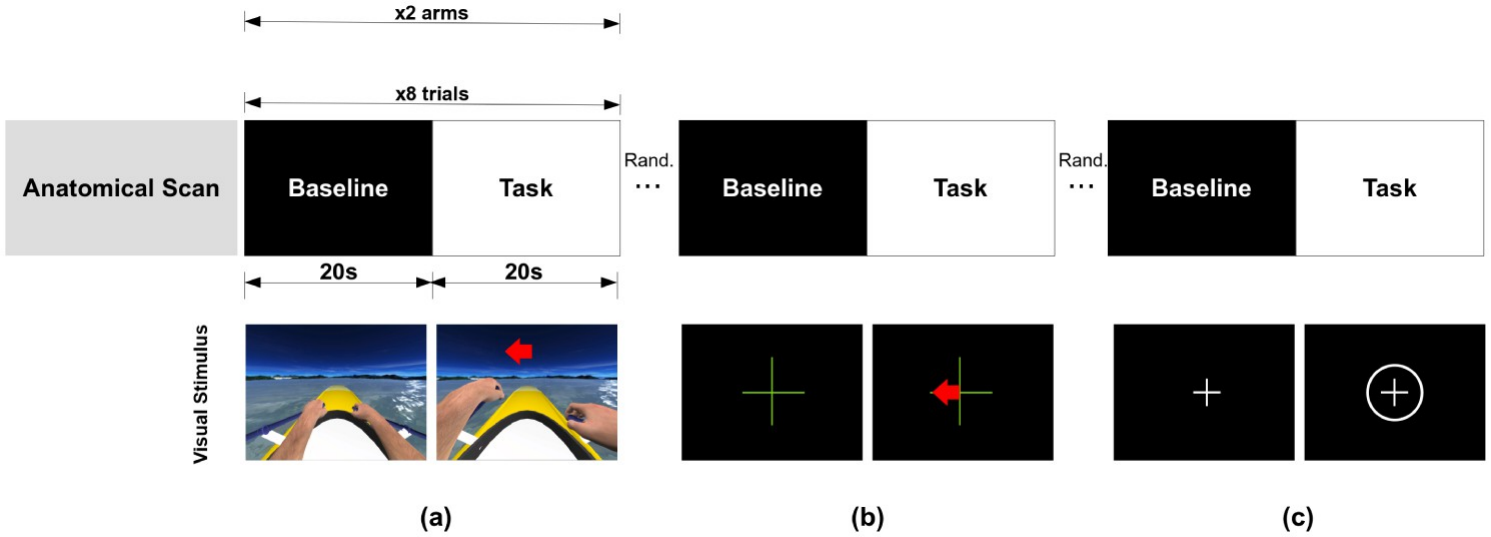
Experimental paradigm. The structure and timings of the trials are shown (top), and the visual instructions are illustrated (bottom), for each task executed by the participants: (a) motor imagery and observation with NeuRow (MI-MO), showing left or right arm movement alternating with no movement in the same scenario; (b) only motor imagery through the Graz paradigm (MI), showing a directional arrow indicating left or right to cue for imagery of left or right arm, alternating including a fixation cross; (c) motor execution (ME), showing a concentric circle around the fixation cross, alternating with the fixation cross.

The following three tasks were performed for left and right arm movement separately, yielding a total of six fMRI runs (pseudo-randomized order): (a) ME: motor execution through finger-tapping; (b) MI: motor imagery only based on the Graz paradigm; and (c) MI-MO: motor imagery with motor observation based on the NeuRow task. Each fMRI run consisted of 8 trials, each with 20 s of baseline followed by 20 s of task (total run duration 5.33 min) (Fig 1).

For the MI-MO condition (NeuRow task), participants were instructed to imagine the kinaesthetic experience of rowing. An adaptation of the originally proposed VR training paradigm [25] was used, whereby participants observed the virtual avatar arm moving while instructed to imagine the movement, but did not control it with their brain activity in a closed-loop (Fig 1(a)).

The MI condition was based on the conventionally used Graz-BCI paradigm [6]. Similar to the MI-MO task, it consisted in imagining the kinaesthetic experience of rowing. However, in this case, a simple directional arrow served as cue (left or right) against an empty black background on the screen with a fixation cross (see Fig 1(b)). The Motor Execution (ME) task consisted in a finger tapping task cued using the appearance of a circle outside a fixation cross (Fig 1(c)).

### Image analysis

The fMRI data were analysed using FSL tools (https://fsl.fmrib.ox.ac.uk/fsl). Standard pre-processing was performed including: non-brain tissue removal using FSL’s BET; motion correction FSL’s MCFLIRT, spatial smoothing using a Gaussian kernel with full width at half maximum (FWHM) of 6.5625 mm, and high-pass temporal filtering with a cut-off period of 100 s. Finally, functional images were normalized to the standard Montreal Neurological Institute (MNI152) T1-weighted image (2x2x2 mm^3^ voxel size) by linear registration using FSL’s FLIRT.

Pre-processed fMRI data were then submitted to a first-level general linear model (GLM) analysis using tool FILM (FMRIB’s Improved Linear Model) [35]. To obtain the explanatory variable (EV) of interest, a boxcar function describing the task paradigm was convolved with the Canonical (Double-Gamma) Haemodynamic Response Function (HRF). Additionally, the 6 motion alignment parameters (3 rotations and 3 translations of the head along the three main axis) were included as confound EV’s of no interest. This GLM was fitted to the data with pre-whitening to correct for temporal autocorrelations. Positive (activation) and negative (deactivation) BOLD changes during the task relative to baseline were assessed as positive / negative parameter estimates for the EV of interest.

### Statistical analysis

Group analysis was then performed using a higher-order mixed-effects GLM. To identify the group average brain activation and deactivation patterns associated with each task, one-sample t-tests were performed for each task and arm (ME Right and Left, MI Right and Left, and MI-MO Right and Left). A 2-way repeated measures ANOVA was used to assess the effects of task (ME vs. MI vs. MI-MO) and arm (Left vs. Right), as well as their interaction. Since a significant effect of task was found, with no interaction with arm, two-sample paired t-tests were then performed for each pair of tasks (ME vs. MI, ME vs. MI-MO, and MI vs. MI-MO), combining right and left arms.

In each case, correction for multiple comparisons was performed by using cluster-extent based thresholding as implemented in FSL (cluster thresholding): p<0.001 (i.e. z>3.1) is used as an initial threshold on the voxel level before data are submitted to cluster thresholding; Gaussian Random Field (GRF) theory is then used to obtain the p value of getting a cluster of a particular spatial extent given the spatial smoothness of the noise in the data and the z threshold used to get the cluster. Finally, we used the probabilistic Harvard-Oxford Cortical [36–39] and Juelich Histological [40–42] atlases to identify brain regions kindred to each activation / deactivation cluster.

## Results

### Average brain activation for each task and arm

**Fig 2:**
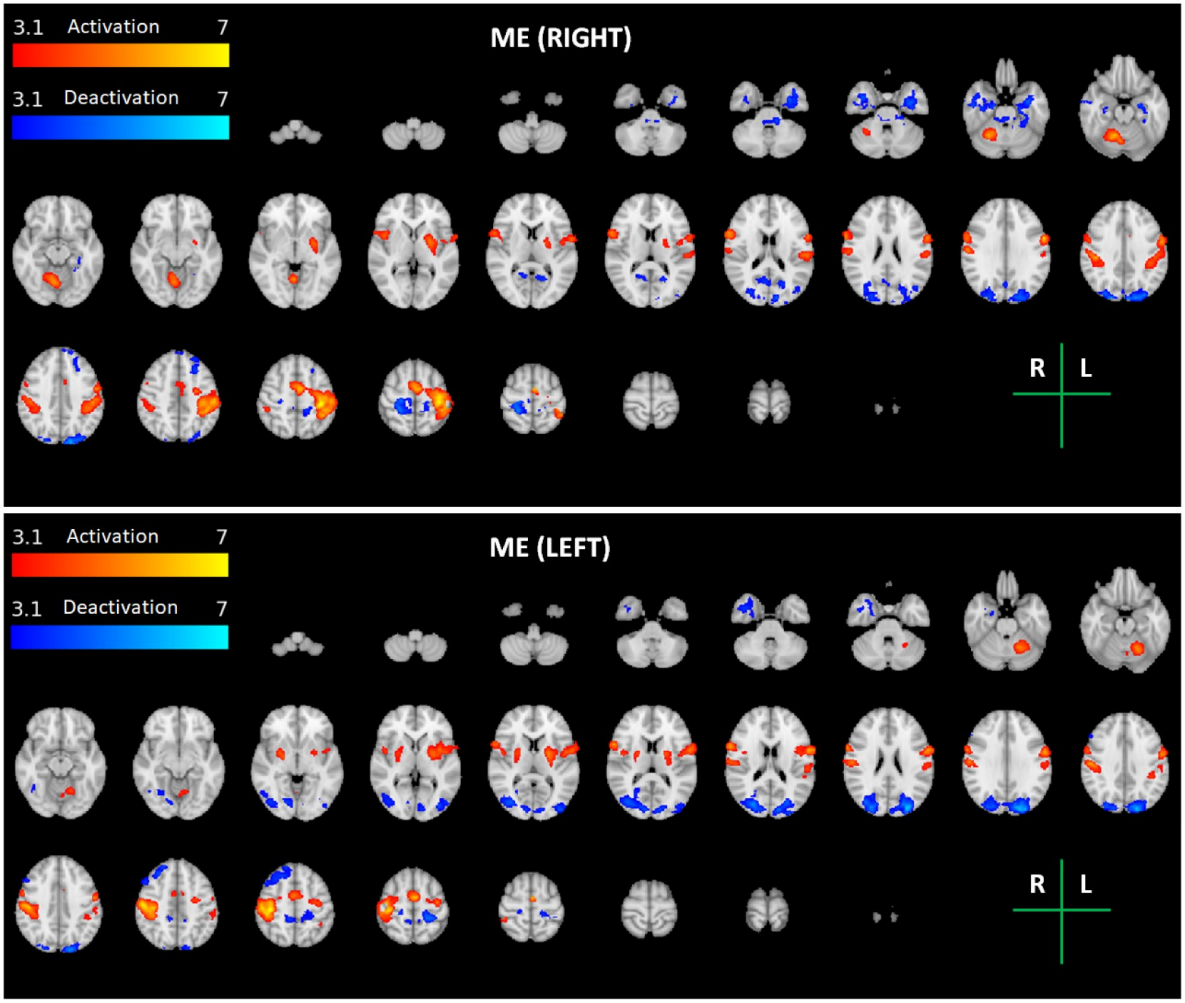
Maps of group activation and deactivation for the motor execution (ME) task for each arm (Right—Top; Left—Bottom). Thresholded z-stat maps of positive (activation) and negative (deactivation) BOLD changes during task vs. baseline (red-yellow and blue-cyan colour scales, respectively) are overlaid on the MNI152 T1-weighted image for a series of representative transverse slices. The brain regions identified in each maps are described in Table 1.

**Fig 3:**
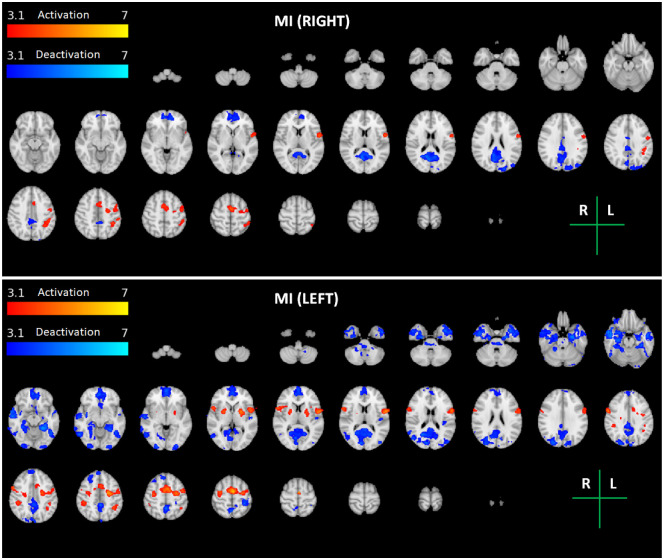
Maps of group activation and deactivation for the motor imagery (MI) task for each arm (Right—Top; Left—Bottom). Thresholded z-stat maps of positive (activation) and negative (deactivation) BOLD changes during task vs. baseline (red-yellow and blue-cyan colour scales, respectively) are overlaid on the MNI152 T1-weighted image for a series of representative transverse slices. The brain regions identified in each maps are described in Table 2.

**Fig 4:**
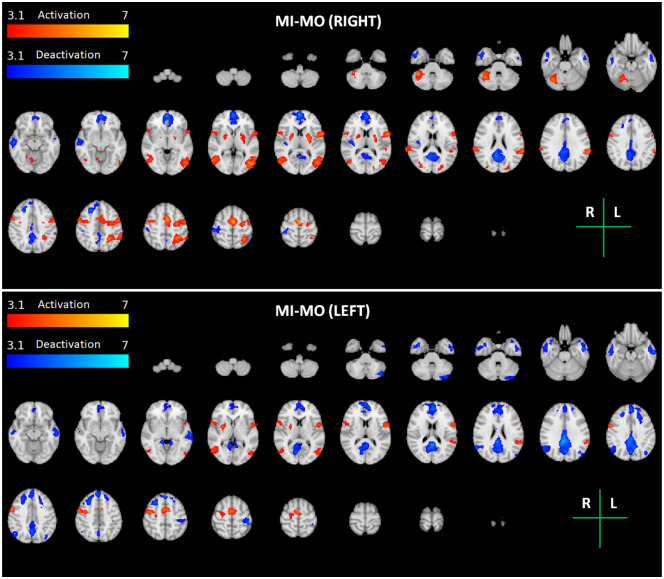
Maps of group activation and deactivation for the motor imagery and observation (MI-MO) task for each arm (Right—Top; Left—Bottom). Thresholded z-stat maps of positive (activation) and negative (deactivation) BOLD changes during task vs. baseline (red-yellow and blue-cyan colour scales, respectively) are overlaid on the MNI152 T1-weighted image for a series of representative transverse slices. The brain regions identified in each maps are described in Table 3.

The group average brain activation and deactivation maps obtained for each task and arm (ME Right and Left, MI Right and Left, and MI-MO Right and Left) are shown in Figs 2–4. The brain activation and deactivation clusters identified in each map are described in Tables 1–3, respectively, including the identified brain areas, as well as their volume, mean and peak z-stat value, and peak MNI coordinates.

**Table 1:** Clusters of group activation and deactivation for the motor execution (ME) task, for each arm (Right and Left).

Brain Region		Volume (*cm*^3^)	Z-score (mean)	Z-score (max)	1—p-val	MNI Peak Coord (mm)		
						X	Y	Z
**Activation: ME (RIGHT)**								
1	Postcentral Gyrus/ Primary Motor Cortex (Right)	45.416	4.24	7.22	>0.9999	-36	-30	52
2	Supramarginal Gyrus, anterior division / Inferior Parietal Lobule (Right)	17.208	3.77	5.7	>0.9999	44	-26	36
3	Cerebellar Lobule V (Right)	10.904	4.09	6.01	>0.9999	14	-58	-18
4	Left Putamen	5.304	3.81	4.98	>0.9999	-24	-2	4
**Activation: ME (LEFT)**								
1	Postcentral Gyrus (Right)	22.704	3.42	7.22	>0.9999	38	-24	50
2	Precentral Gyrus (Left)	20.200	3.87	5.66	>0.9999	-58	2	18
3	Cerebellar Lobule VI (Left)	6.766	3.82	5.39	>0.9999	-18	-58	-22
4	Precentral Gyrus (Right) / Broca’s Area BA44	6.736	3.83	5.41	>0.9999	62	10	18
5	Parietal Operculum Cortex (Left) / Secondary Somatosensory Cortex	5.680	3.37	4.14	>0.9999	-56	-24	18
6	Justapositional Lobule Cortex/ Premotor Cortex	4.384	4.00	5.88	>0.9999	-2	-6	60
7	Right Pallidum	3.704	3.58	4.69	0.9985	22	-8	-4
8	Precentral Gyrus (Left) / Premotor Cortex	2.936	3.65	4.93	0.9948	-38	-14	60
**Deactivation: ME (RIGHT)**								
1		12.608	3.68	5.76	>0.9999	-14	-86	40
2	Temporal Fusiform Cortex, Anterior Division/ Gray Matter Hippocampus entorhinal cortex (Left)	12.032	3.48	4.69	>0.9999	-32	-2	-36
3	Occipital Pole/Visual Cortex V2	6.920	3.56	5.33	>0.9999	22	-88	34
4	Precentral Gyrus/Corticospinal Tract	5.680	3.45	4.51	>0.9999	40	2	-30
5	Postcentral Gyrus/Primary Somatosensory Cortex (Right)	4.344	3.80	5.16	0.9997	24	-38	60
6	Superior Frontal Gyrus	3.440	3.39	4.32	0.9987	-26	24	44
7	Precuneous Cortex/ Visual Cortex V1 (Left)	3.024	3.38	3.99	0.9972	16	-56	10
8	Primary Somatosensory Cortex (Left)	1.024	3.43	4.25	0.9532	-18	-36	54
**Deactivation: ME (LEFT)**								
1	Lateral Occipital Cortex, Superior Division	22.464	3.66	5.3	>0.9999	26	-84	22
2	Lateral Occipital Cortex, Superior Division	21.28	3.86	6.59	>0.9999	-26	-86	26
3	Postcentral Gyrus/Primary Somatosensory Cortex (Left)	5.696	3.54	4.67	>0.9999	-20	-36	58
4	Middle Frontal Gyrus	5.672	3.46	4.79	>0.9999	28	30	48
5	Temporal Pole	3.312	3.38	4.31	0.9718	40	4	-38

**Table 2:** Clusters of group activation and deactivation for the motor imagery (MI) task, for each arm (Right and Left).

Brain Region		Volume (*cm*^3^)	Z-score (mean)	Z-score (max)	1—p-val	MNI Peak Coord (mm)		
						X	Y	Z
**Activation: MI (RIGHT)**								
1	Central Opercular Cortex, Secondary Somatosensory Cortex	10.112	3.48	4.36	>0.9999	-56	0	8
2	Supramarginal Gyrus, Anterior Division/ Inferior Parietal Lobule	5.536	3.44	4.39	>0.9999	-50	-38	54
3	Juxtapositional Lobule Cortex/ Premotor Cortex	5.168	3.55	4.73	>0.9999	-2	-4	60
**Activation: MI (LEFT)**								
1	Juxtapositional Lobule Cortex/ Premotor Cortex	26.456	3.86	6.27	>0.9999	-2	-4	58
2	Precentral Gyrus (Right)/ Premotor Cortex	9.704	3.59	5.10	>0.9999	-58	4	36
3	Left Putamen	3.160	3.79	5.01	0.9982	-24	2	2
4	Supramarginal Gyrus, Posterior Division	2.752	3.48	4.47	0.9961	-40	-48	48
5	Supramarginal Gyrus, Anterior Division (Right)	2.656	3.5	4.22	0.9954	44	-34	42
6	Right Putamen	1.808	3.67	4.94	0.9982	24	4	4
**Deactivation: MI (RIGHT)**								
1	Precuneous Cortex/Visual Cortex V2	28.52	3.64	5.05	>0.9999	4	-66	18
2	Paracingulate Gyrus,	8.240	3.52	4.71	>0.9999	8	54	0
3	Lateral Occipital Cortex, Superior Division	6.888	3.45	4.64	>0.9999	-20	-90	24
**Deactivation: MI (LEFT)**								
1	Middle Temporal Gyrus, posterior division	105.752	3.59	5.49	>0.9999	52	-10	-18
2	Parahippocampal Gyrus, posterior division	56.232	3.60	5.39	>0.9999	-24	-42	-14
3	Occipital Pole/Visual Cortex V3V (Right)	3.304	3.51	4.41	0.9986	28	-92	-6
4	Occipital Pole/Visual Cortex V3V (Left)	3.224	3.51	4.64	0.9984	-28	-94	-6
5	Postcentral Gyrus/Primary Somatosensory Cortex (Left)	2.120	3.55	4.51	0.9866	-32	-36	58
6	Heschl’s Gyrus/Primary auditory cortex	1.808	3.39	4.21	0.9742	-54	-14	2
7	Cerebellar Lobule VIIIb (Right)	1.744	3.41	4.62	0.9703	24	-44	-46

**Table 3:** Clusters of group activation and deactivation for the motor imagery and observation (MI-MO) task, for each arm (Right and Left).

Brain Region		Volume (*cm*^3^)	Z-score (mean)	Z-score (max)	1—p-val	MNI Peak Coord (mm)		
						X	Y	Z
**Activation: MI-MO (RIGHT)**								
1	Juxtapositional Lobule Cortex (Premotor Cortex)	17.352	3.79	5.88	>0.9999	-2	-4	60
2	Superior Parietal Lobule (Left)/ Precuneus Cortex	14.960	3.70	5.49	>0.9999	-28	-52	50
3	Lateral Occipital Cortex, Inferior Division (Left)	11.192	3.78	5.51	>0.9999	-44	-74	4
4	Right Crus I	10.112	3.65	5.51	>0.9999	36	-56	-30
5	Lateral Occipital Cortex, Inferior Division (Right)/ Visual Cortex V5	9.200	3.66	5.08	>0.9999	56	-62	4
6	Precentral Gyrus (Right)/ Broca’s Area BA44	5.952	3.72	5.01	>0.9999	-58	6	10
7	Precentral Gyrus (Right)/ Premotor Cortex	4.200	3.43	4.74	0.9998	38	-4	50
8	Lateral Occipital Cortex, superior division (Left)	3.880	3.58	4.96	0.9996	-24	-8	8
9	Supramarginal Gyrus, Posterior Division (Right)/ Inferior Parietal Lobule	2.912	3.38	4.99	0.9979	72	-38	20
10	Central Opercular Cortex	2.240	3.48	4.28	0.9917	50	8	4
11	Right Putamen	2.040	3.44	4.66	0.9872	26	-4	6
12	Lateral Occipital Cortex, Superior Division (Right)	1.528	3.55	3.99	0.9592	-24	-82	22
**Activation: MI-MO (LEFT)**								
1	Precentral Gyrus (Right)/ Premotor Cortex	6.792	3.66	5.65	>0.9999	56	2	38
2	Juxtapositional Lobule Cortex/ Premotor Cortex	5.184	3.82	5.07	>0.9999	6	0	54
3	Precentral Gyrus (Right), Broca’s Area BA44	4.168	3.68	4.84	>0.9999	-56	4	16
4	Lateral Occipital Cortex, Inferior Division (Right)	3.640	3.50	4.32	0.9989	54	-64	2
5	Supramarginal Gyrus, Posterior Division (Left)	2.840	3.50	4.75	0.9956	-56	-46	24
6	Lateral Occipital Cortex, Inferior Division (Left)	2.488	3.47	4.16	0.9920	-50	-76	6
7	Inferior Frontal Gyrus, Pars Opercularis (Right)	2.240	3.40	4.04	0.9871	56	14	2
8	Right Putamen	1.696	3.52	4.37	0.9613	24	6	2
**Deactivation: MI-MO (RIGHT)**								
1	Cingulate Gyrus, posterior division	26.168	3.74	5.22	>0.9999	6	-46	34
2	Frontal Pole	24.256	3.68	5.44	>0.9999	2	60	8
3	Middle Temporal Gyrus, posterior division	6.408	3.57	5.15	>0.9999	60	-20	-12
4	Postcentral Gyrus/Primary Somatosensory Cortex (Right)	2.680	3.55	4.63	0.9966	30	-36	66
5	Middle Temporal Gyrus, anterior division	2.112	3.55	4.5	0.9890	-54	-6	-20
6	Central Opercular Cortex/Secondary somatosensory cortex (Right)	1.624	3.65	5.02	0.9673	40	-18	16
**Deactivation: MI-MO (LEFT)**								
1	Cingulate Gyrus, posterior division/ Cingulum (Right)	37.472	3.92	5.79	>0.9999	4	-44	28
2	Paracingulate Gyrus	34.016	3.57	4.98	>0.9999	-2	48	20
3	Middle Temporal Gyrus, anterior division	10.264	3.58	4.96	>0.9999	-58	-10	-24
4	Middle Temporal Gyrus, posterior division	6.992	3.53	4.62	>0.9999	56	-20	-18
5	Angular Gyrus/Inferior parietal lobule Pga (Right)	6.424	3.55	4.6	>0.9999	60	-56	24
6	Crus I (Left)	3.600	3.54	4.34	0.9989	-40	-82	-34
7	Postcentral Gyrus/Primary Somatosensory Cortex (Left)	2.896	3.55	4.84	0.9962	-44	-36	60
8	Lateral Occipital Cortex, superior division	1.872	3.36	4.14	0.9731	-40	-70	36

There is consistent activation of motor and premotor areas across the three tasks. For ME, both the contralateral primary motor cortex and cerebellum, as well as the premotor cortices, are clearly activated. For MI and MI-MO, the Juxtapositional Lobule Cortex, corresponding to the supplementary motor area (SMA), is consistently activated, as well as the putamen. Compared with both ME and MI, MI-MO recruits additional brain regions, namely the occipital and parietal cortices and the inferior frontal gyrus, yielding an overall greater volume of brain activation. In terms of the arm side, the expected lateralization of brain activity is observed, with greater activation of the contralateral hemisphere. The lateralization is greater for the right relative to the left arm tasks, also as expected. The observed lateralization patterns are less clear for the MI task when compared with the ME and MI-MO tasks.

We found significant deactivation of the ipsilateral primary somatosensory cortex in all tasks and runs (except MI with the right arm, where it did not reach statistical significance after multiple comparison correction). We also found clear deactivation of regions belonging to the default mode network during the motor imagery and observation tasks (MI and MI-MO) but not the motor execution task (ME).

### Differences in brain activation between tasks and arms

**Fig 5:**
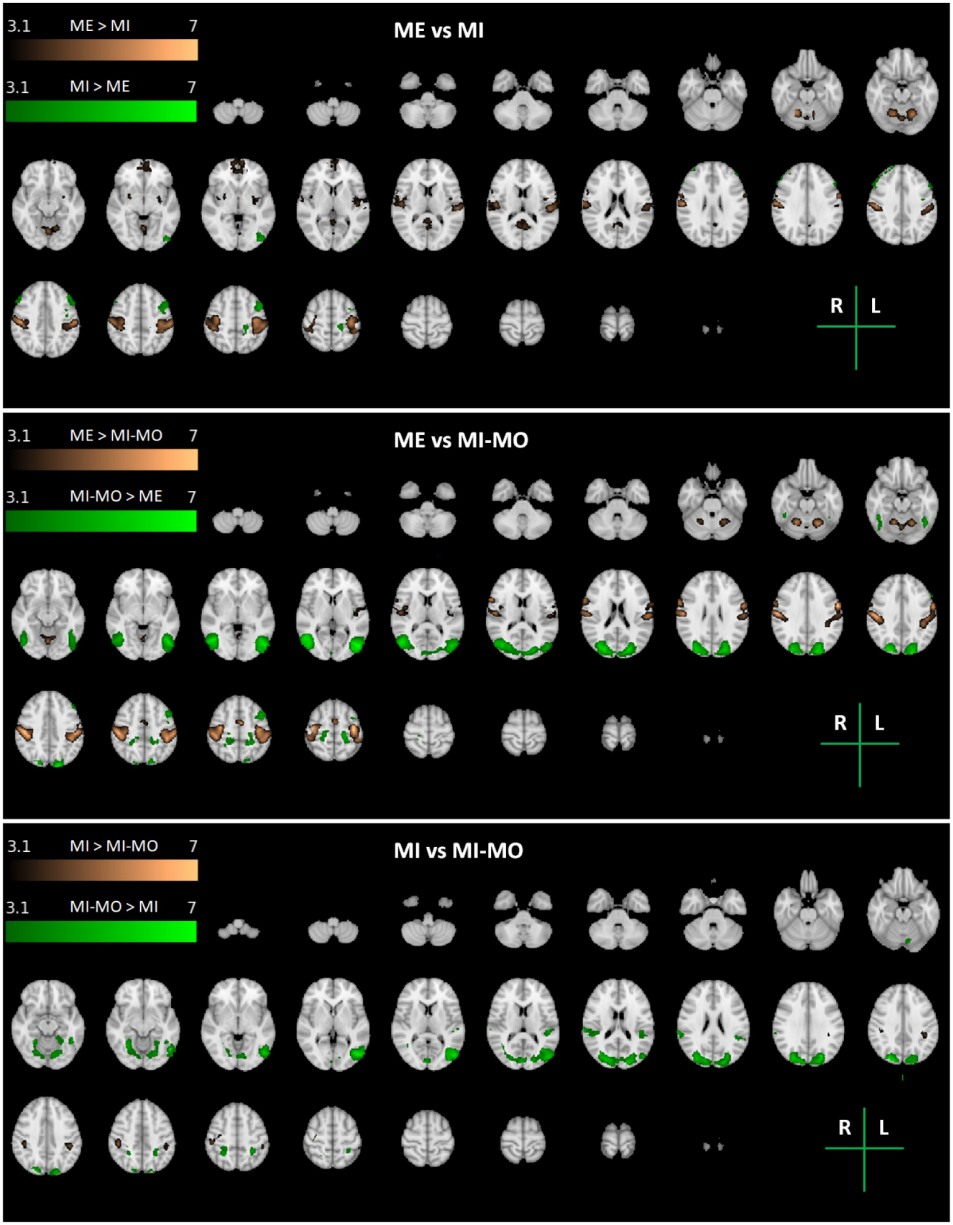
Maps of group activation differences between tasks. Pairwise t-tests between tasks, across both arms (Right and Left): MI vs. ME (top); MI-MO vs. ME (middle); and MI vs. MI-MO (bottom). Thresholded z-stat maps (colour scales) are overlaid on the MNI152 T1-weighted image for a series of representative transverse slices. The brain regions identified in each map are described in Table 4.

The 2-way repeated measures ANOVA yielded significant main effects of task and arm, with no significant interactions between them. The maps of significant differences between pairs of tasks (MI-MO vs. MI, MI-MO vs. ME, and MI vs. ME) are presented in Fig 5. The brain regions identified in each map are described in Table 4, including the identified brain areas, as well as their volume and peak activation z-stat value and MNI coordinates. As expected, the ME task more strongly activated the primary motor and sensorimotor cortices, as well as the cerebellum, when compared to both imagery tasks, MI and MI-MO. Interestingly, a few areas were also more strongly activated by the MI and MI-MO tasks than ME. While MI further activated small areas of the frontal and occipital cortices, MI-MO produced greater activation over large areas across the occipital and parietal cortices. This was also evident when directly comparing MI-MO with MI.

**Table 4:** Clusters of group activation differences between tasks. Two-sample paired t-tests between tasks, across both arms (Right and Left): ME vs. MI; ME vs. MI-MO; and MI vs. MI-MO.

Brain Region		Volume (*cm*^3^)	Z-score (mean)	Z-score (max)	1—p-val	MNI Peak Coord (mm)		
						X	Y	Z
**ME > MI**								
1	Postcentral Gyrus (Right)	23.064	3.93	6.1	>0.9999	50	-20	38
2	Precentral Gyrus (Left) / Primary Motor Cortex	15.512	4.21	5.94	>0.9999	-34	-20	54
3	Cerebellar Lobule VI (Right)	14.552	3.72	5.52	>0.9999	24	-56	-24
4	Parietal Operculum Cortex (Left)	8.848	3.60	5.03	>0.9999	-52	-24	14
5	Frontal Pole (Left)	6.232	3.44	4.54	>0.9999	-8	68	2
6	Precentral Gyrus (Left)/ Primary Motor Cortex	1.656	3.72	5.06	0.9729	-60	0	32
**MI > ME**								
1	Middle Frontal Gyrus (Left)	5.912	3.56	4.75	>0.9999	-44	6	52
2	Frontal Pole	2.544	3.34	4.04	0.9955	20	56	36
3	Lateral Occipital Cortex, Inferior Division	1.848	3.67	4.76	0.982	-44	-82	-4
4	Postcentral Gyrus (Left)/Primary Somatosensory Cortex	1.520	3.61	4.39	0.9634	-18	-32	54
**ME > MI-MO**								
1	Precentral Gyrus / Premotor Cortex (Left)	29.408	4.29	7.35	>0.9999	-58	0	32
2	Postcentral Gyrus (Right)/ Inferior Parietal Lobule Pft (Right)	27.720	4.41	6.96	>0.9999	50	-20	38
3	Cerebellar Lobule VI (Left)	10.280	3.94	5.69	>0.9999	-18	-58	-22
4	Juxtapositional Lobule Cortex/ Premotor Cortex BA6	2.008	3.97	5.85	0.9771	-4	-6	54
**MI-MO>ME**								
1	Lateral Occipital Cortex, Inferior Division/ Visual Cortex V5 (Left)	70.704	4.35	7.31	>0.9999	-48	-76	4
2	Precentral Gyrus (Left)/ Premotor Cortex BA6	3.880	3.79	4.95	0.9991	-18	-26	60
3	Precentral Gyrus / Corticospinal Tract (Right)	3.560	3.52	4.96	0.9985	20	-26	60
4	Middle Frontal Gyrus (Left)/ BA44	3.176	3.69	4.71	0.9972	-44	10	50
**MI > MI-MO**								
1	Supramarginal Gyrus, Anterior Division (Right)	2.880	3.67	5.02	0.9979	44	-32	44
2	Supramarginal Gyrus, Anterior Division (Left)	1.920	3.46	4.3	0.9861	-44	-34	40
**MI-MO > MI**								
1	Lateral Occipital Cortex, Superior Division/ Lateral Occipital Cortex Inferior Division / Visual Cortex V5	41.976	4.14	6.40	>0.9999	-44	-74	4.0
2	Temporal Occipital Fusiform Cortex, Occipital Fusiform Gyrus/ Visual Cortex V4	7.240	3.45	4.55	>0.9999	30	-56	-10
3	Supramarginal Gyrus, Posterior Division, Planum Temporale, Parietal Opercular Cortex (Left)	2.784	3.65	5.33	0.9975	-48	-30	12
4	Supramarginal Gyrus, Posterior Division, Planum Temporale, Parietal Opercular Cortex (Right)/ Inferior Parietal Lobule	2.760	3.55	4.75	0.9974	68	-34	24
5	Superior Parietal Lobule (Left)/ Primary Somatosensory Cortex	1.608	3.68	5.03	0.9973	-28	-44	54
6	Superior Parietal Lobule (Right)/ Primary Somatosensory Cortex	1.400	3.42	4.04	0.9557	26	-52	52

## Discussion

In this study, we showed that we could elicit stronger brain activation with our newly developed NeuRow task, combining MI and MO in a more ecologically-valid scenario, when compared with a conventional MI task based on the Graz BCI paradigm using abstract instructions. Specifically, when compared to the abstract MI task (MI), as well as to an overt motor execution task (ME), NeuRow (MI-MO) recruited a large volume of the brain across the occipital and parietal cortices, additionally to the motor and premotor cortices.

### Recruitment of sensorimotor cortex

The ME task strongly activated the primary motor and somatosensory cortices, as well as cerebellar structures, namely cerebellar lobules I-IV, VI and VIII. There is evidence supporting a topographic organization of the cerebellum, with lobules V-VI and VIII involved in motor processing [43]. Moreover, resting-state functional connectivity studies report correlation of activity in sensorimotor cortices with activity in cerebellar lobules V, VI and VIII [44–46]. Therefore, our findings support that the recruited cerebellar regions are kindred to motor processing, more precisely with a finger tapping movement.

In turn, although with less extension, both imagery tasks, Graz (MI) and NeuRow (MI-MO), elicited activity in sensorimotor areas as well, as expected [11]. A more detailed observation shows that the volume recruited by MI-MO and MI centers mainly in premotor regions, which are typically associated to action preparation [47]. These findings are in line with converging evidence of consistent recruitment of brain regions typically linked to sensorimotor behaviour across ME, MI and MO tasks [10, 28].

In fact, despite the large overall brain activation differences between the MI-MO and MI tasks, we found no significant differences in the activation of premotor regions. This supports the idea that both tasks are able to effectively promote MI and activate the brain areas involved in action preparation [48]. The fact that these neuronal correlates are also partly shared with the execution of the movement indicates that both MI paradigms are in principle adequate for neurorehabilitation. This is particularly true considering that neuroplastic changes that lead to the recovery of function after stroke are thought to involve the premotor cortex [49].

### Recruitment of additional brain areas

When compared with the MI task, the MI-MO task further activated areas of the parietal cortex consistent with the MNS [12]. This may be explained by the fact that, unlike Graz MI, NeuRow involved the observation of the arm movement in addition to imagery. In particular, we found significantly greater activation of the inferior parietal lobule (IPL), namely in its sub-regions PFcm, PF, and PFt [50]. A previous study suggested that the combined activity of these IPL sub-regions with sensorimotor activation may play a specific role in visuospatial and attention-based motor processing [51]. On the other hand, the IPL PFt sub-region has been proposed to be the human homologue of the PF region in primates, which is supposed to contain mirror neurons [28]. These findings corroborate the activation elicited by NeuRow given its motor observation component.

Regarding the greater activation found in the occipital cortex with MI-MO compared with MI, it is probably the result of a combination of several factors. In general, it is not surprising that greater visual activation is induced by MI-MO given the greater visual content of the rowing scenario compared with the MI task. More specifically, the fact that the visual stimulus consists of a first-person perspective of one’s own arm rowing a moving boat on a lake may explain the activation of area V5 of the primary visual cortex, which is known to be involved in the processing of visual motion [52]. Moreover, we also found greater activation of area V4 of the primary visual cortex, which is thought to integrate information from areas V1 and V2 [53]. However, several studies have shown that V4 neurons may also be directly involved in the processing of a wide range of properties of visual stimuli, including surface properties (colour, shape, texture), movement of the visualized object, and even visual attention [53–55]. This function is also consistent with the execution of the MI-MO task.

Moreover, greater activation of the precuneus was also found. This brain area has been reported to be involved in a wide spectrum of visual tasks, including visuospatial imagery, episodic memory retrieval, and first-person perspective, all of which are present in the NeuRow task [56].

Overall, our findings agree with previous work showing that a combination of MI with MO leads to stronger activation of the brain than either condition alone [17, 18]. Moreover, the more ecologically-valid environment of NeuRow may contribute to a stronger engagement of various brain areas involved in different aspects of the task, ranging from visual attention to motor preparation and observation. An overall greater engagement of the brain may be desirable in rehabilitation settings, since it is likely associated with improved focus and motivation. In fact, these are essential to enhance adherence to therapy and, thus, rehabilitation outcomes [57].

### Brain deactivations

We found deactivation of the primary somatosensory cortex (S1) mostly in the ipsilateral hemisphere in all tasks. This is partly in agreement with a recent study that reported deactivation of the primary motor cortex (M1) during motor imagery [58]. However, a region of interest (ROI) analysis was performed and only the M1 and the SMA mean BOLD signals were analysed, not the whole brain voxelwise as in our case. Therefore, it is possible that S1 deactivation also occurred in their study. On the other hand, we could have also detected M1 deactivation had we performed a similar ROI analysis. Hence, it is possible that deactivation of both the primary motor and somatosensory cortices occurs to some degree during MI. Regarding the deactivation of the early visual cortex with MI, this is probably the result of a demanding task involving another sensory modality. Indeed, previous studies have reported crossmodal deactivations in sensory cortices which are irrelevant to the task at hand, during the performance of mental imagery [59].

Interestingly, we also found deactivation of regions belonging to the default mode network (DMN) during both motor imagery tasks (MI and MI-MO) but not the motor execution task (ME). These results are consistent with the observation that the DMN is suppressed during the execution of cognitively demanding, goal-directed tasks [60], and suggest that participants are cognitively engaged when attempting to imagine moving more than when overtly executing the movement. It is also highly relevant for the envisaged applications in stroke rehabilitation that decreased DMN connectivity has been previously reported in stroke patients [61]. Interestingly, a recent study showed that interactions between the DMN and the sensorimotor network facilitated neuroplasticity in stroke rehabilitation [62].

### Limitations

One possible confound during the execution of motor imagery tasks is that participants may perform subtle muscle contractions [63]. Since we did not record the EMG concurrently with our fMRI acquisitions, we cannot completely rule out this possibility. Nevertheless, the fact that we did not observe M1 activation during the motor imagery tasks suggests that participants were correctly following the instructions and were not contracting their muscles. Similarly to a recent study addressing this issue by discussing the absence of M1 activation [58], we therefore believe muscle contraction is not a confound in our experiment.

During the MI-MO condition, the displayed stimulus (NeuRow) is from a 3D rendering of a virtual environment. Although it is not projected to the subject’s eyes stereoscopically, it provides a first-person perspective of the virtual body during a motor task. By ecological validity, we refer to the extent to which the training task can be generalized and applied to real-world situations. In the context of stroke rehabilitation, ecological validity is important to ensure that the training and interventions provided to stroke patients reflect the challenges they face in their everyday lives. In the case of upper-limb rehabilitation, we initially prioritize the training of proximal movements, hence the design of the task (NeuRow) was targeted at training shoulder and arm movement through a rowing action.

The fact that not all expected brain activations and deactivations were found for every run in our study indicates that it lacked statistical power in some cases. This was particularly notorious in the case of the right arm MI, which yielded an activation and deactivation pattern that was considerably different from that of the left arm MI. Most activations and deactivations were in fact present when analysing the results without correcting for multiple comparisons but failed to survive such correction. Nevertheless, the statistical power of the study was sufficient to find significant differences between tasks, which was the main goal and novelty of our study.

### Future work

Our results show that specific areas of the somatomotor cortex are both activated and deactivated during motor imagery tasks. The utilization of VR-based neurofeedback could be designed to target such specific brain regions, or neural networks, and reinforce them in a closed-loop through the use of a BCI [58, 64]. The more informed selection of the target region, potentially in a personalized approach, could help optimize the effectiveness of neurofeedback—BCI systems and improve neurorehabilitation outcomes.

To obtain results that are more closely relevant to the desired clinical application, future research should investigate brain activity elicited by these different paradigms in an older population and also in a population of post-stroke survivors undergoing an appropriate rehabilitation intervention. Furthermore, future studies should investigate which of the reported brain regions most contribute to the neuroplastic changes that occur after training with the tool. This could allow the design of tailored MI/MO-driven ecologically-valid VR-BCI systems to achieve greater rehabilitation outcomes.

To confirm our hypothesis that VR-based neurofeedback / BCIs may promote better efficacy, future research should address this question by comparing MI-MO-based VR-BCI strategies with conventional MI paradigms in post-stroke rehabilitation interventions.

## Conclusion

We showed that an ecologically-valid task combining motor imagery and observation can recruit sensorimotor neural systems as well as brain structures involved in visual processing and attention-based motor tasks. This work extends previous research on brain activation during concurrent MI and MO tasks, by further including a realistic scenario and in this way providing increased ecological validity. The enhanced brain activation highlights the potential of such MI-MO tasks to be used in VR-based BCI systems for stroke rehabilitation interventions.
